# On Topical Medication of the Larynx

**Published:** 1856-01

**Authors:** 


### ON TOPICAL MEDICATION OF THE LARYNX.

The profession well know that Dr. Green, of New York, claims to have introduced the sponge probang through the larynx, trachea down the bronchia, and quite into the cavities from which tuberculous matter had been discharged.

This was gravely doubted by many, for very cogent reasons; whereupon a committee was chosen for the purpose of determining the truth of Dr. Green's declarations, that he had, and could, accomplish it. After a careful examination, and numerous closely conducted experiments, the majority of the committee reported unfavorably to the proposition. Since the rendition of this report, there has been kept up a discussion on the subject, with some manifestations of warmth.

Since then John Erichsen, Prof. of Surgery at University College and Surgeon to the Hospital, London, has published an article of recent date, which is found in a recent number of the "American Journal of the Medical Sciences," in which he takes strong grounds against even the probability of such a thing, sustaining his views with the most conclusive arguments. We think this will prove a *sequiter* to the discussion of this subject, for we regard his arguments as unanswerable.

